# Untangling biodiversity interactions: A meta network on pollination in Earth's most diverse tropical savanna

**DOI:** 10.1002/ece3.11094

**Published:** 2024-03-11

**Authors:** Ludmilla M. S. Aguiar, Ugo M. Diniz, Igor D. Bueno‐Rocha, Laura R. A. Filomeno, Luísa S. Aguiar‐Machado, Priscilla A. Gomes, Pedro H. B. Togni

**Affiliations:** ^1^ Departamento de Zoologia, Instituto de Ciências Biológicas Universidade de Brasília Brasília DF Brazil; ^2^ Chair of Plant‐Insect Interactions, School of Life Sciences Technical University of Munich Freising Germany; ^3^ Programa de Pós‐Graduação em Ecologia, Instituto de Ciências Biológicas Universidade de Brasília Brasília DF Brazil; ^4^ Programa de Pós‐Graduação em Zoologia, Instituto de Ciências Biológicas Universidade de Brasília Brasília DF Brazil; ^5^ Departamento de Ecologia, Instituto de Ciências Biológicas Universidade de Brasília Brasília DF Brazil

**Keywords:** Brazilian Cerrado, conservation hotspot, floral visitors, modularity, mutualistic network, trait matching, zoophilia

## Abstract

Pollination is vital for ecosystem functioning, especially in biodiversity‐rich regions like the Brazilian Cerrado. Our research establishes a comprehensive meta network of pollinator–plant interactions within this biome. We quantified the importance of different pollinator groups, identifying keystone species. We examined potential biases in sampling effort and the spatial behavior of interactions within the heterogeneous Cerrado plant physiognomies. Our investigation uncovered 1499 interactions among 293 plant species and 386 visitor species, with legitimate pollination accounting for 42.4% of the interactions. The network exhibited modularity, driven by bees and insects, with vertebrates bridging diurnal and nocturnal modules. While a generalized pattern emerged, high specialization existed within modules due to habitat diversity. Bees, particularly *Apis mellifera* (exotic) and *Trigona spinipes* (native), played central roles as network hubs. Hummingbirds and bats, engaged in specialized interactions showing strong connectivity within and between modules. Interestingly, invertebrate–vertebrate modules were more prevalent than expected in the meta network. However, a bias was evident, primarily within specific biogeographical districts with fragmented landscapes and intrusion from other biomes. Variations in plant species and endemism rates influenced pollinator occurrence and the Cerrado network topology. Our study offers valuable insights into pollinator–plant interactions within the Cerrado, encompassing both invertebrates and vertebrates. The modeled network represents a significant step in understanding the structural complexity of pollination networks, integrating partial networks from diverse pollination systems within heterogeneous habitats. Nevertheless, a biogeographical bias could limit a comprehensive understanding of network functionality across the Cerrado.

## Graphical abstract

We addressed how pollination meta network with vertebrate and invertebrate species is assembled in the world's most diverse savanna and identified potential biogeographical bias within the region. We found a generalization of interactions at the meta network level but a high degree of specialization within the modules, where bees are the module connectors and vertebrates account for most specialized interactions. We found a solid biogeographical bias that limits our knowledge of how interactions are assembled in this severely threatened biome.

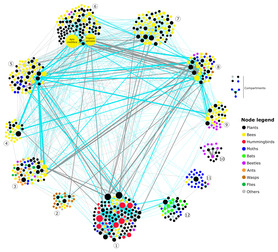

## 1 INTRODUCTION

Pollination and seed dispersal are critical interactions shaping plant communities, with far‐reaching implications for biodiversity and ecosystem services worldwide (Ballesteros‐Mejia et al., 2016; Gelmi‐Candusso et al., 2017; Neuschulz et al., 2016; Ramos et al., 2020; Samways et al., 2020). Animal‐mediated pollen dispersal is widely recognized as a key driver of plant population structure, often exceeding the importance of seed dispersal (Calviño‐Cancela et al., 2012; Gamba & Muchhala, 2020; Kartzinel et al., 2013; Nazareno et al., 2021; Valenta et al., 2017). Although ecological interaction network studies have provided invaluable insights into community organization globally, they exhibit a significant bias toward non‐tropical regions (Olesen et al., 2007; Schleuning et al., 2012).

Agricultural expansion has resulted in the conversion of 23 Mha, equivalent to 15% of the world's savanna hotspots (Hu et al., 2020; Rausch et al., 2019). At the same time, most studies on pollinators in the tropics have provided limited comprehensive insight into the complexities of pollination–pollinators–plant relationships (Vizentin‐Bugoni et al., 2018). However, tropical ecosystems are not only critical biodiversity hotspots but they also play a pivotal role in global climate regulation, agricultural productivity through pollination services, and the preservation of unique species with potential applications in various industries (Costanza et al., 2014; Sutton & Costanza, 2002). Consequently, unraveling the intricacies of tropical ecosystems becomes paramount for understanding global biodiversity patterns, ecosystem functioning, and ensuring agricultural stability. Recent estimates indicate substantial untapped potential of bees and vertebrates as crop pollinators in Brazil, because 88.4% and 95.2% of the species in these groups, respectively, compose the neglected diversity of potential crop pollinators in the country (Lopes et al., 2021). By exploring these uncharted interactions between plants and pollinators, essential ecological connections can be uncovered, enriching our understanding of tropical ecosystem resilience and their contributions to sustaining life on Earth.

The Cerrado, a Brazilian biodiversity hotspot (Myers et al., 2000), is an ideal study system to gain insight into the organization of plant–pollinator networks in tropical regions. Despite experiencing significant loss of native vegetation, the Cerrado still harbors more than 12,000 plant species, with approximately 4000 being endemic (Flora e Funga do Brasil, 2024; Mendonça et al., 2008). Cerrado's floral richness is represented primarily by Leguminosae, Compositae, Orchidaceae, Gramineae, Rubiaceae, Melastomataceae, Myrtaceae, Euphorbiaceae, Malpighiaceae, and Lythraceae, accounting for 51% of its flora (Mendonça et al., 2008). This diversity is distributed across seven biogeographic districts, each characterized by distinct climates and species composition, resulting in varied landscapes within the Cerrado's geographic range (Françoso et al., 2020).

Additionally, the Cerrado, located in central Brazil, at the heart of the country, is bordered by the Amazon Forest to the north, the Atlantic Forest to the south and southeast, and the Caatinga to the northeast. Spanning approximately 1,500,000 square kilometers, it ranks as the second largest biome in South America and its extensive latitudinal and altitudinal variations result in remarkable environmental and habitat diversity. Savannas dominate the landscape, covering approximately 72% of the region. However, the presence of forested areas interwoven within this savanna backdrop imparts a mosaic‐like appearance to the region (Cardoso Da Silva & Bates, 2002). The flat plateau summits, ranging from 500 to 1700 m above sea level, are adorned with semi‐deciduous to evergreen savanna‐like vegetation (Eiten, 1990). Another unique plant physiognomy associated with rocky outcrop plateaus is the *campos rupestres*, renowned for its highly endemic flora (Eiten, 1990). This exceptional heterogeneity contributes to the Cerrado's remarkable species richness and endemism (Klink & Machado, 2005).

Despite the substantial knowledge available on Cerrado plants, further research on animal–plant interactions, particularly on ecosystem services such as seed dispersal and pollination, is warranted (Ramos et al., 2020). Seasonal restrictions can impact plant reproduction and pollinator availability of pollinators (Gottsberger, 1986; Martins et al., 2021; Silberbauer‐Gottsberger & Gottsberger, 1988). However, a comprehensive understanding of the overall organization of these interactions is lacking, as most pollination studies focus on specific plant or animal groups, plant physiognomie groups, or strata, resulting in limited knowledge about the general patterns of these interactions.

The extinction and coextinction of species pose a significant threat to plant–pollinator networks (Assunção et al., 2022). Therefore, it is crucial to better understand the resilience of these networks and the geographic variability in their vulnerability, particularly in biodiversity hotspots like the Cerrado. Liu et al. (2021) demonstrated that the phylogenetic diversity is relatively susceptible to the loss of tropical and high‐latitude continental plants compared to other regions, emphasizing the need to bridge this knowledge gap not only for a deeper understanding of macroevolutionary processes but also to develop appropriate management strategies to preserve biodiversity in this fragmented biome (Castilla et al., 2017; Hadley & Betts, 2012; Rabeling et al., 2019).

Against this backdrop, our study aimed to investigate the structure of pollinator/flower visitor–plant interactions in the Brazilian Cerrado biome and identify potential geographic biases that hinder a comprehensive understanding of how these interactions are established within the biome. We conducted a comprehensive literature review encompassing invertebrate and vertebrate pollinators' interactions with flowering plants in natural Cerrado environments. We later constructed a meta network to model these interactions. Our specific aims were to address the following questions: (i) Which pollinator groups exhibit the highest centrality and connectivity? (ii) How has the incorporation of geographic gaps at the biome and local scales, particularly within distinct plant phisiognomies contexts, influenced connectivity? Our study is designed to offer a critical perspective on the network's structure and the pollination services rendered by invertebrates and vertebrates in the Cerrado. This input contributes to a broader comprehension of tropical ecosystems and their ecological dynamics.

## 2 METHODS

### 2.1 Review of the literature and mapping of study localities

In December 2022, an extensive literature search was conducted to gather comprehensive information on pollination in the Cerrado biome. The research was centered on the interactions between flowers and both vertebrate and invertebrate animals, with a specific emphasis on the significance of different pollinator groups and spatial connectivity. Native plant species were exclusively considered, while instances of pollination in exotic plants, articles related to agricultural areas, and articles that did not specifically address animal‐mediated pollination were excluded from the analysis. We avoided agricultural areas and exotic plant species because they may often have artificial and managed pollination practices, involving human interventions such as hand pollination or the use of domesticated pollinators such as honeybees. Including such articles could introduce confounding factors and deviate from the study's emphasis on natural animal‐mediated pollination interactions within the Cerrado biome. By excluding these articles, we focused on the ecological dynamics of pollinator–plant interactions in the natural context of the Cerrado. Nevertheless, we kept the articles evaluating the interactions of naturally occurring honeybees with Cerrado plants because, although exotic, this species is well established in natural areas and is part of the community of interacting species in the biome. Web of Science, Scielo, and Google Scholar were the searched platforms to retrieve relevant scientific papers. In Web of Science, the following keywords were used: “pollinat*,” “Cerrado,” “savannah,” or “savanna,” and “Brazil.” In Scielo, the keywords “pollination,” “pollinator,” “savanna,” “Cerrado,” and “Brazil” were employed. Articles in both English and Portuguese languages were considered. To enhance the search, a back‐and‐forth citation search was conducted for each identified article to identify additional relevant articles. Backward citation searches ensured the inclusion of foundational and historically significant works, while forward citation searches helped capture the latest developments and current discussions in the field. Combining these approaches, we ensure that the research is based on a thorough examination of the available literature.

Inclusion in our analyses was based on the explicit observation of pollination or flower visitation by animals, or when such interactions could be inferred from the text. To ensure the native status of the plants discussed in the articles, their life habits and occurrence records were cross‐referenced using the Flora do Brasil Portal (Flora e Funga do Brasil, 2024; Table S1). For the preparation of maps, records with vague or unspecified geographic information regarding pollination interactions were excluded. Only records with specific and well‐defined geographic positions at the country level were included, treating each locality as an independent case. This approach enabled us to accurately plot the data on the Cerrado floral district map according to Françoso et al. (2020).

### 2.2 Network structure

We constructed a binary interaction matrix (a_ij_) based on a coalescence of many different data obtained from the literature to construct the meta network using an approach similar to Nascimento et al. (2020). The matrix represented the interactions between the lower level plant species (i) and the higher‐level floral visitors (j). This matrix encompassed all interactions reported in the studies, including legitimate pollination events and visitation interactions where pollination was not explicitly assessed. True pollination events were assigned by the authors of the studies we reviewed and imply that pollen was effectively transferred between flowers resulting in fruit formation. Floral visitors were all other animals that visited a plant flower, but there is no evidence or observation that pollen was effectively transferred, nor that reproduction occurred. Using this approach, we separated truly mutualistic interactions made by pollinators from visitation events. To prevent redundancy within the same genera or families, we only included species identified to the species level, which also accounted for identifications with “aff.” and “cf.” We did not include interactions where the species were identified as “sp.” and “spp.” to avoid merging different species with potentially distinct traits.

### 2.3 Modularity and connectivity analyses

We examined whether the network exhibited a typical structural pattern observed in mutualistic networks—modularity. Modularity refers to the formation of subgroups within the community where species interactions are more frequent within the subgroups compared to interactions with the rest of the network. In pollination networks, modularity is associated with ecological specialization between plant and floral visitor groups (Olesen et al., 2007), and in networks containing several animal or plant groups, modules are frequently correlated with pollination syndromes (Danieli‐Silva et al., 2012). Here, we sought to understand whether the biome‐wide network shows a significant network structure and if modules are correlated with pollinator groups. We assign the plant syndromes as they were considered by the authors in the papers we reviewed.

We estimated Barber's modularity (QB) using a simulated annealing optimization algorithm implemented in the software MODULAR, which is well‐suited for large datasets (Marquitti et al., 2014). The QB values range from zero to one, with one indicating a perfectly modular network. To assess the significance of modularity, we compared the observed QB value to a theoretical benchmark generated by a null model (null model 2) embedded in MODULAR (Bascompte et al., 2006; Marquitti et al., 2014). The null model created 1000 random networks based on the original matrix while preserving the observed number of links per node. The significance (*p*‐value) was calculated as the probability that the random matrices produce a modularity value equal to or greater than the observed QB.

### 2.4 | Assessing the importance of floral visitors in the network

We categorized floral visitors into major taxonomic groups, that is, bees (Hymenoptera), wasps (Hymenoptera), ants (Hymenoptera), beetles (Coleoptera), moths (Lepidoptera), flies (Diptera), hummingbirds (Aves: Trochilidae) and bats (Chiroptera). Rarer pollinators, including non‐Trochilidae birds, thrips, and nonvolant mammals, were grouped under the category “other.” The aim was to analyze the importance of these pollinator groups in terms of centrality and their potential as connectors within the network.

We calculated three species‐level metrics to evaluate the centrality of each pollinator species in the network to assess trends in overall importance in the network across pollinator taxonomical groups. For each floral visitor, we measured (i) their degree (*d*), or simply the number of interacting partners; (ii) closeness centrality (CC), which measures the number of shortest paths crossing a specific node and thus how central it is in the graph in relation to others, serving as a proxy for relevance (Martín González et al., 2010); and (iii) betweenness centrality (BC), which measures the frequency with which a node lies on paths between other nodes, thus reflecting its relevance as a connector between different parts of the network (Newman, 2003). These species‐level indices, which represent different aspects of a species' role in the network, were grouped by pollinator taxonomical group, and variations among groups were assessed using generalized linear models (GLM). Four models were run, one for each metric as a dependent variable, and floral visitor taxonomic group as an independent variable. A Gaussian error distribution was assumed for species' degree (continuous variable), while a quasibinomial error distribution was assumed for closeness and betweenness centralities (proportions). One‐way ANOVAs were used to assess significance, and Tukey post hoc tests were performed to determine pairwise differences between floral visitor groups within the GLMs. All inferential analyses were performed in the R Software (R Core Team, 2023). Species‐level indices were calculated with the bipartite package.

### 2.5 Distribution of interactions across the Cerrado vegetational mosaic

Due to the strong heterogeneity and patchy nature of the Cerrado with its various vegetation types (i.e., plant physiognomies), we aimed to understand how well‐represented each vegetation type is in terms of sampling effort, as well as how interconnected are plant–pollinator interactions are among the different vegetation types. For this purpose, data were collected from each article found in our revision in which specific type of vegetation physiognomy interactions was sampled. These are categorized as in Oliveira‐Filho and Ratter (2002), which include the typical plant physiognomies found in the Cerrado, and can be grouped roughly into (i) typical Neotropical savannas (cerrado *sensu stricto*, *cerradão* or arboreal savanna, and other vegetations associated with these, such as palm swamps and rupestrian cerrado), (ii) open vegetations or grasslands (e.g., *campo limpo*, *campo sujo*, rupestrian grasslands), and (iii) forests (riparian, gallery, and semidecidual forests). Data on these plant physiognomies were collected as it was stated in each specific article. In rare cases where authors did not specify the vegetation type any further than the generic term “Cerrado,” we assumed that the work was conducted in a typical savanna formation (cerrado *sensu stricto*). With these data, we grouped the number of interactions sampled per larger vegetation type (typical savannas, open environments, and forests) and per specific plant physiognomy within each category to assess the distribution of the research effort in the biome and to uncover potential gaps.

Additionally, we also sought to understand how well connected the larger vegetation categories are with each other, to understand how well pollinators serve as ecological bridges between plants found only in each vegetation type. We thus classified each plant node according to where the species was found in the published papers, that is, savanna‐, grassland‐, or forest‐exclusive, occurring in more than one category (savanna–forest, savanna–grassland, or forest–grassland), or occurring in all categories. For articles focused on identifying pollinators that did not specify the types of plant phisiognomies to which the plant belonged, this information was gathered from the literature and in the Flora Brasil database (see Section 2.1).

In order to measure how well connected the vegetation types are to each other, we measured the connectance (the proportion of realized links in relation to the total possible number) and the mean degree (averaged number of partners per species) for three subnetworks containing only species found in two pairs of vegetation categories (savannas and grasslands only, savannas and forests only, and forests and grasslands only), as well as for the entire network to be used as a benchmark. The spatial networks, connectance, and mean degree values were also obtained in Gephi (Bastian et al., 2009).

## 3 RESULTS

### 3.1 Plant–pollinator interactions and network structure

The literature‐based search yielded 312 articles, and of these, 122 studies specifically examined interactions between pollinators and native plant species in the Cerrado biome. Among these studies, the majority (79.5%) focused on plant reproductive biology and pollination of floral visitors, while the remaining 20.5% aimed at understanding animal behavior during floral visits or pollination.

The modeled interaction network included a total of 386 animal species (75 families) and 292 plant species (73 families), resulting in 2693 reported interactions between animal and plant families. The dominant pollinator family in the network was Apidae (Hymenoptera), accounting for 15% of all interactions with plants. Halictidae (9.3%) and Megachilidae (4.0%) were the next most prevalent bee families. Hummingbirds (Aves: Trochilidae) and bats (Chiroptera: Phyllostomidae) were significant vertebrate pollinators in the network, representing 8.2% and 4.0% of the interactions, respectively. Regarding plants, the families most reported that interact with floral visitors were Rubiaceae (10.2%), Malpighiaceae (9.3%), Arecaeceae (6.5%), Melastomataceae (5.7%), and Fabaceae (5.4%). In particular, a proportion of families (20.6%) exclusively interacted with vertebrate pollinators, such as Loranthaceae, Acanthaceae, Amaryllidaceae, and Combretacea.

We identified pollination syndromes in the Cerrado biome based on 1267 reviewed interactions. The most common syndromes were melittophily (55.64% of records), ornithophily (17.13%), and chiropterophily (7.73%). Other syndromes included sphingophily (3.79%), cantharophily (2.29%), and myophily (1.03%). Anemophily, myrmecophily, and psicophily together accounted for 0.24% of the records. Additionally, 7.26% of the interactions were generically attributed to entomophily, and 4.86% were classified as a generalist syndrome (unspecified).

**FIGURE 1:**
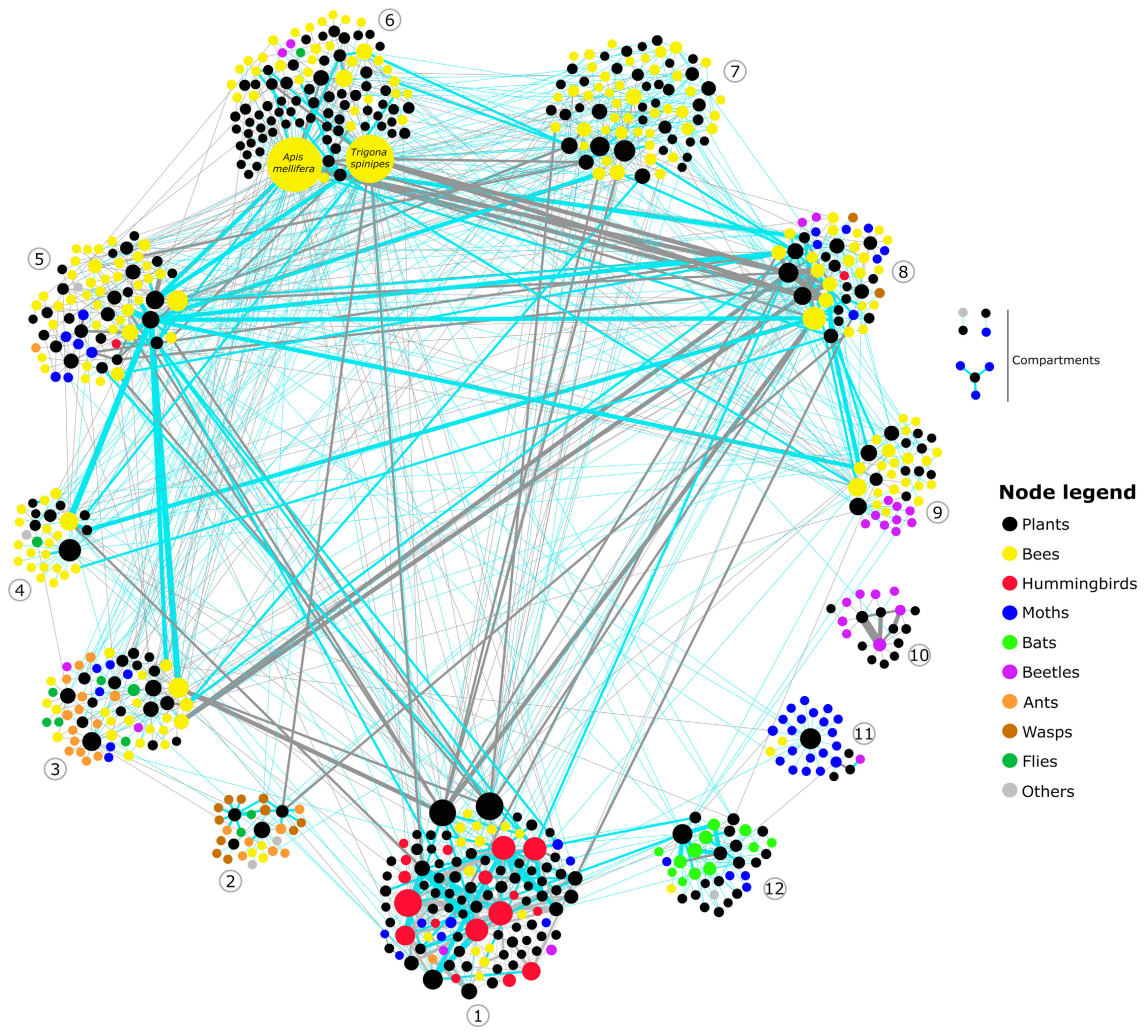
Species‐level interaction network between plants and floral visitors in the Brazilian Cerrado. Species clusters correspond to modules optimized by the Barber algorithm (see Section 2: Methods). Black nodes correspond to plants, and colored nodes represent different groups of floral visitors. Node size is proportional to species degree (number of partners). Interactions are classified as either legitimate pollination (blue edges) or floral visitation (gray edges), where pollination was not assessed.

Using data up to the species level, a final network was modeled, consisting of 1499 unique plant–pollinator interactions (Figure 1). Legitimate pollination interactions represented 42.4% of all interactions in our data, represented by 201 animal species and 155 plant species. Bees were the most frequent floral visitors (64.8%), followed by lepidopterans (13.3%), vertebrates (bats and hummingbirds, 7.7%), wasps (5.7%), and beetles (5.2%). Ants, flies, and other flower visitors (non‐Trochilidae birds, thrips, Hemiptera, and rodents) represented 7.8% of the flower visitors.

Structurally, the network was significantly modular, presenting a moderately high modularity (QB = 0.60, *p* < .0001), suggesting that the network was divided into distinct modules or clusters of species that were more densely connected within their respective modules (Figure 1). The network exhibited a phylogenetic pattern, with certain groups of pollinators (e.g., hummingbirds, bats, beetles, wasps, and lepidopterans) predominantly grouped together in the same or closely related modules. Bees, on the contrary, were versatile in their floral interactions and were present in multiple modules, except for one module exclusively composed of beetle species. Two species of bees, the exotic African honeybee (*Apis mellifera*) and the native stingless bee (*Trigona spinipes*), shared a module and stood out as highly central pollinators and were involved in a greater number of interactions compared to other species, which is visually perceptive in the network (Figure 1).

### 3.2 Degree and centrality of species

**FIGURE 2:**
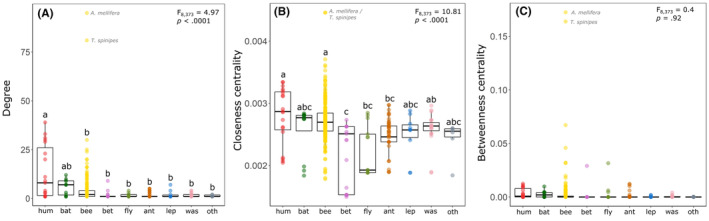
Variations in the scores of species‐level indices of the various floral visitor groups in the Cerrado meta network. A: degree (*d*), B: closeness centrality (CC), and C: betweenness centrality (CC). Pollinator groups: hummingbirds (hum), bats (bat), bees (bee), beetles (bet), ants (ant), lepidopterans (lep), flies (fly), wasps (was), and others (oth). Insets contain the results of statistical analyses, and the letters above boxplots represent significance groups as determined by the post hoc analysis. The outlying and central bees *Apis mellifera* and *Trigona spinipes* were highlighted in all graphs.

In terms of importance, *A. mellifera* and *T. spinipes* achieved very high and outlying values of degree (*d* = 95 and *d* = 81, respectively) and closeness centrality (CC = 0.00446 for both), suggesting the interaction with a surprisingly high number of plant species across the entire network (Figure 2a,b). However, due to the very variable nature of the group in terms of number of partners and a high frequency of peripheral species, bees had an overall low degree value in the network, comparable to other arthropod groups (Figure 2a). Hummingbirds again stood out as the most prolific species, significantly differing from arthropod groups in terms of degree, closely followed by bats (Figure 2a). Closeness centrality also varied significantly across groups, although with a less clear pattern (Figure 2b). Hummingbirds were on average more central than other groups, although slightly, and differed significantly, alongside bees, from very peripheral groups such as beetles, flies, and wasps (Figure 2b). Other prolific floral visitor species with many partners were mostly hummingbirds, such as *Chlorostilbon lucidus* (*d* = 39), *Amazilia fimbriata* (*d* = 33), *Phaethornis petrei* (*d* = 31), and *Thalurania urcate* (*d* = 30). Other highly central floral visitors included mostly large bees, such as *Eulaema nigrita* (CC = 0.0037), *Bombus morio* (CC = 0.0036), and *Oxaea flavescens* (CC = 0.0035), and the eusocial bee species *Paratrigona lineata* (CC = 0.0036). Betweenness centrality did not vary significantly across floral visitor groups (Figure 2c). Here again, *A. mellifera* and *T. spinipes* acted as highly connective species across the network (BC = 0.16 and BC = 0.17, respectively). Other species with high BC values worth highlighting include, again, larger bees (*E. nigrita*, BC = 0.067; *B. morio* = 0.032) and eusocial stingless bees (*Trigona hyalinata*, BC = 0.046; *P. lineata*, BC = 0.032).

Highly influential plant species in the network included typical and emblematic species of the Cerrado, such as the bee‐pollinated Vochysiaceae *Qualea parviflora* (*d* = 39, CC = 0.0052) and *Q. multiflora* (*d* = 37, CC = 0.0051), the bat‐pollinated Caryocaraceae *Caryocar brasiliense* (*d* = 23, CC = 0.0049), and the hummingbird‐pollinated Gesneriaceae *Sinningia elatior* (*d* = 23, CC = 0050). In terms of relevance as connectors, the same four species presented the highest BC values (*C. brasiliense*, BC = 0.086; *S. elatior*, BC = 0.070; *Q. parviflora*, BC = 0.057; *Q. multiflora*, BC = 0.049), alongside the beetle‐pollinated Araceae *Philodendron mello‐barretoanum* (BC = 0.069).

### 3.3 Distribution of interactions across the Cerrado mosaic

**FIGURE 3:**
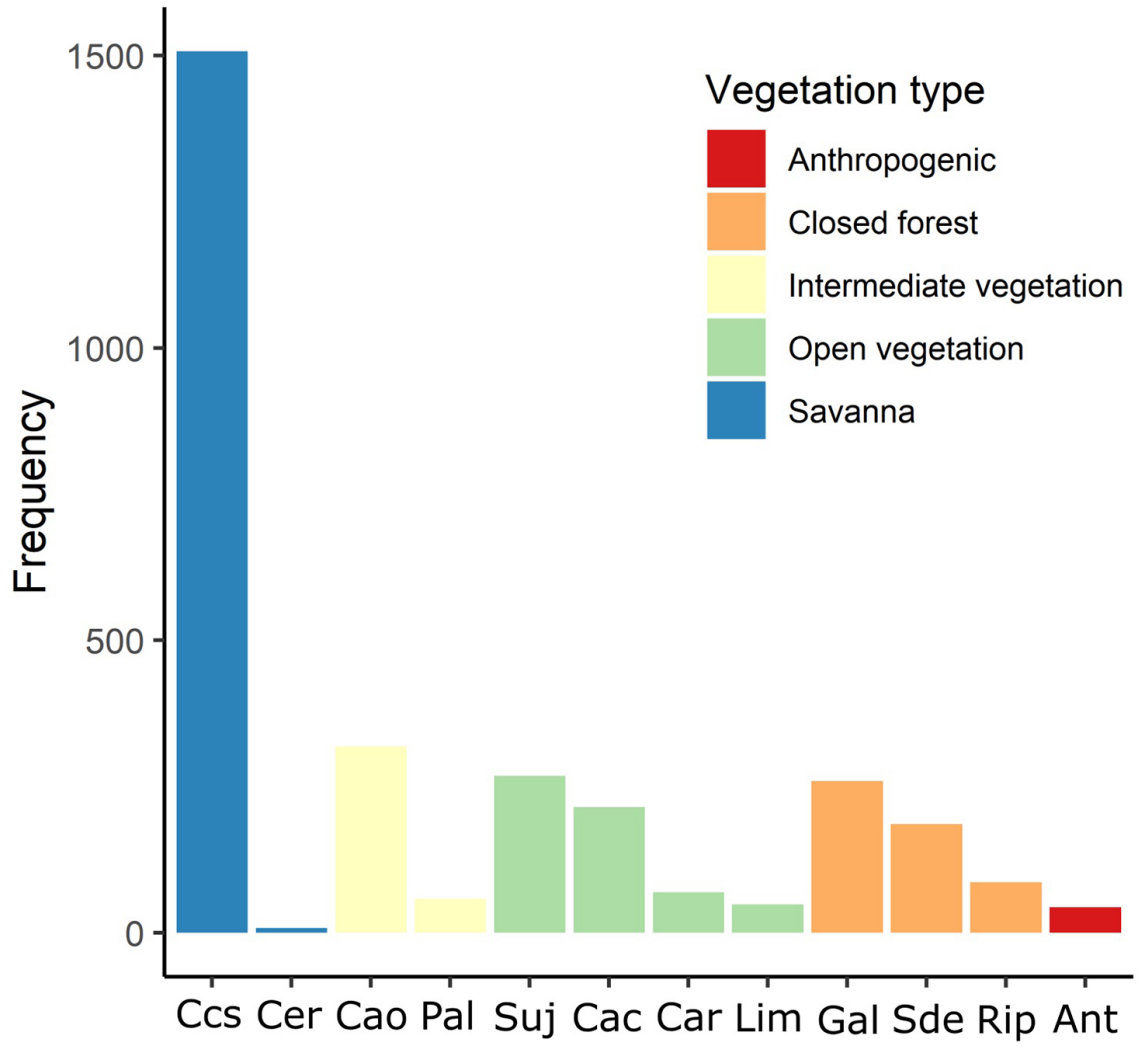
The distribution of interactions reported by the reviewed articles according to vegetation categories: savannas, intermediate vegetations related to savannas, open vegetation, forests, and anthropogenic areas. Specific physiognomies: Css—cerrado *sensu stricto*; Cer—rupestrian cerrado; Cao—*cerradão* (arboreal savanna); Pal—palm swamps; Suj—*campo sujo* (grassland with a sparse occurrence of bushes and trees); Cac—*campo cerrado* (grassland with a high occurrence of bushes); Car—*campo rupestre* (grassland with rocky outcrops); Lim—*campo limpo* (regular grassland); Gal—gallery forest; Sde—semidecidual forest; Rip—riparian forest; Ant—Anthropogenic (deforested areas, farms, or cities).

Roughly half of all interactions reported from the articles (49.4%) were sampled in the cerrado *sensu stricto* vegetation type, the typical and emblematic plant physiognomy that defines most of the Cerrado (Figure 3). Open vegetations, which encompass various types of grasslands, and forest formations normally found along rivers such as gallery and riparian forests, were less represented in comparison to savannas and accounted roughly for 20% of interactions reported each (Figure 3).

**FIGURE 4:**
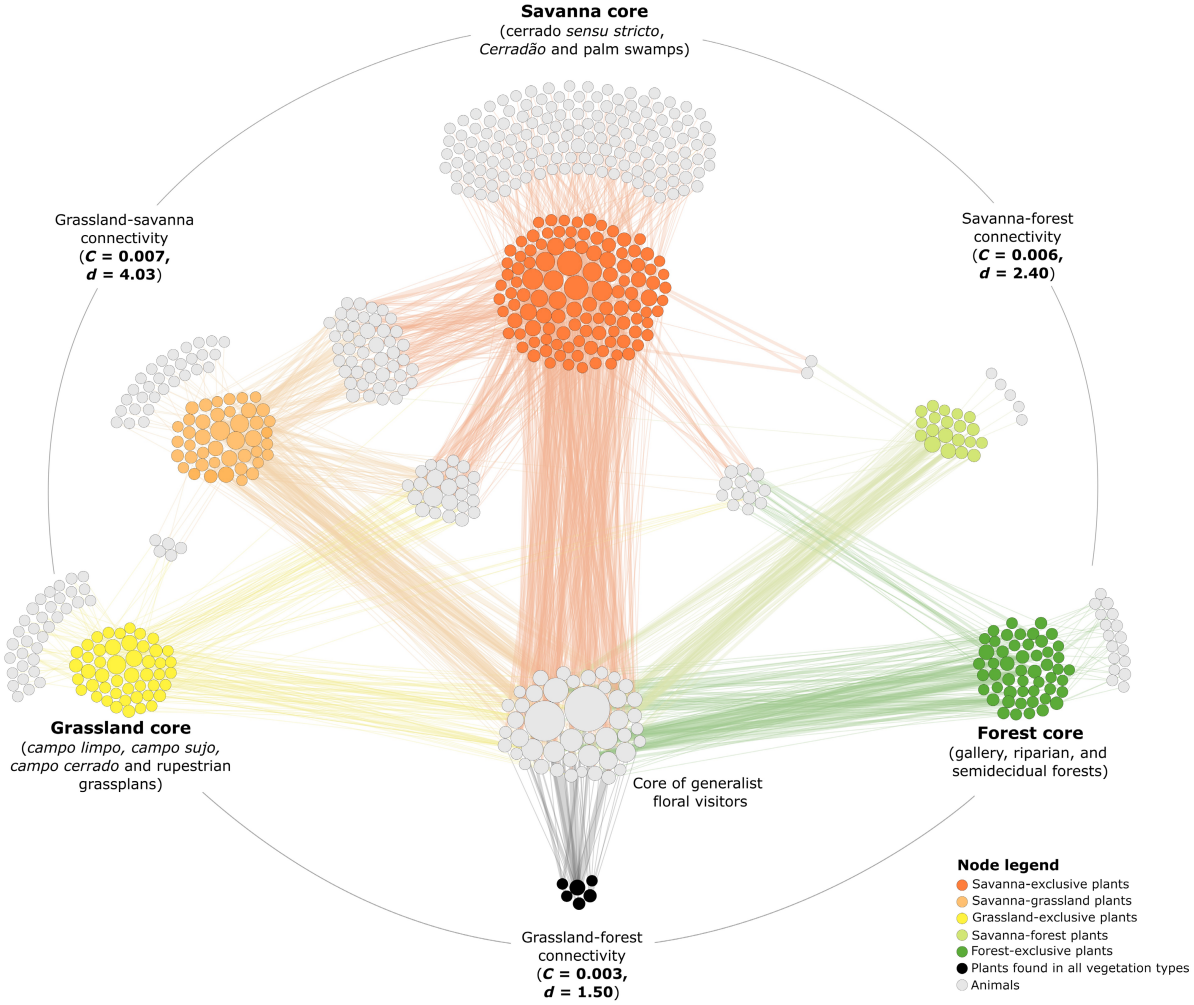
Binary bipartite interaction network between floral visitors and plants in the Brazilian Cerrado, with the subdivision of plant species according to their occurrence in the major vegetation categories found in the biome. Node size corresponds to degree, and edge size to the number of times that the interaction was recorded in the literature. Inset values between vegetation cores correspond to the connectance (*C*) and mean degree (*d*) for the subnetworks containing only the pair of vegetation types in question.

The overrepresentation of the savanna vegetation type is easily observed in a spatially explicit network (Figure 4). Apart from the 118 plant species reported exclusively within savannas (38% of all plant species), 158 flower visitor species (41% of all floral visitors) visited savanna‐exclusive plants only. This proportion was much lower for open vegetation types (34% or 9%) and forests (17% or 4%). Despite this concentration of interactions within savannas, the different plant physiognomies were relatively well connected, particularly due to a core of highly generalist floral visitors that visited plants in all vegetation types. This core contained 12% of all visitors in the network and encompassed the prolific *A. mellifera* and *T. spinipes*. The full network showed a connectance of 0.007 and a mean degree of 4.4, while the savanna–grassland subnetwork was the most connected and approached these values (Figure 4). Connectivity decreased at the savanna–forest subnetwork and reached the lowest values at the forest–grassland subnetwork, with less than half of the connectance and mean degree of the entire network (Figure 4).

### 3.4 Geographical distribution of interactions within the biogeographic districts of the Cerrado

**FIGURE 5:**
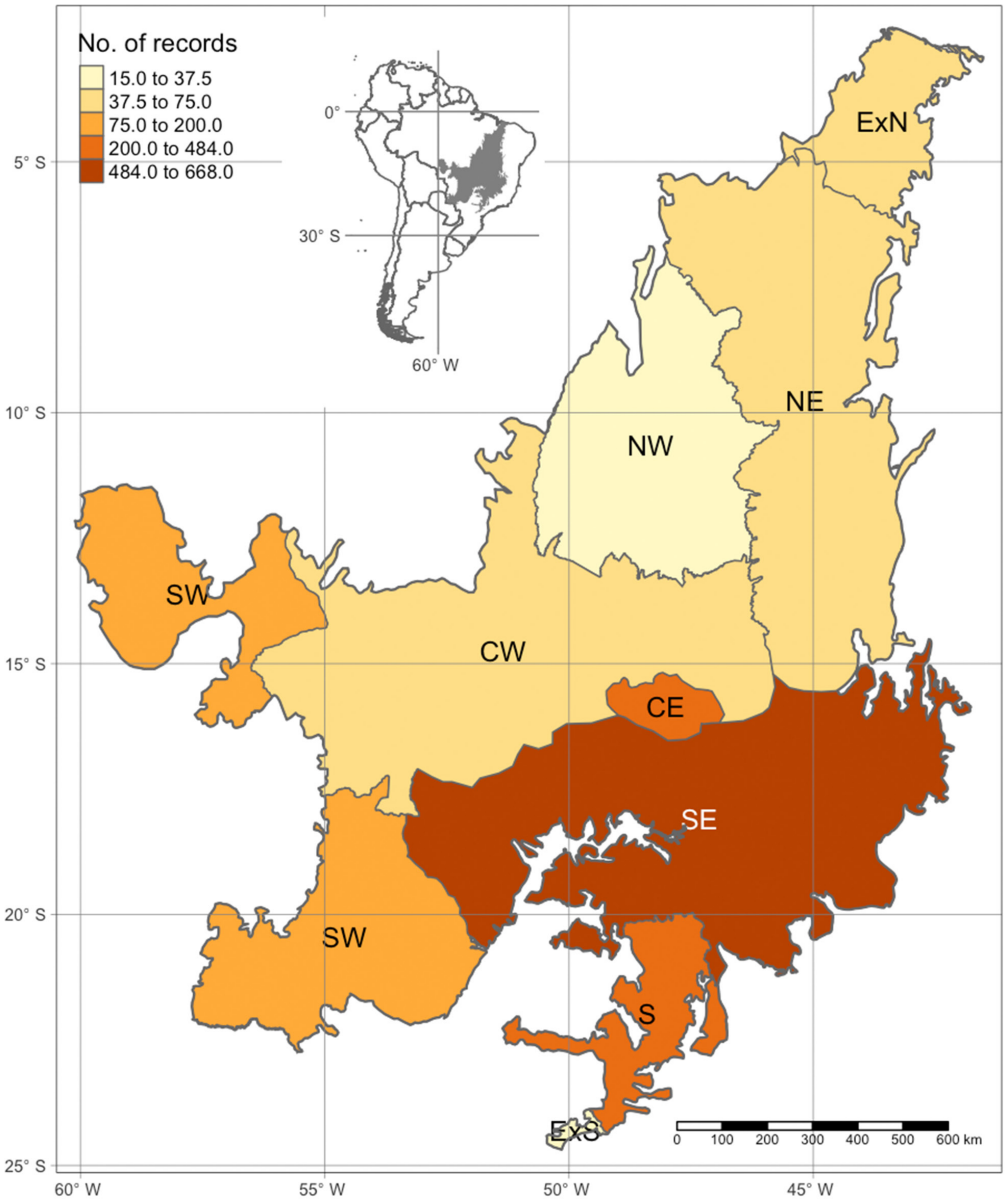
Records of flower‐visiting/pollinator–plant interaction studies in the seven biogeographic districts of the Cerrado biome in Brazil, determined by Françoso et al. (2020)), based on the differences in plant species composition, endemism, and climatic conditions among biogeographic districts. CE—Central; CW—Central‐west; NE—North‐East; NW—North‐west; S—South; SE—South‐East; SW—South‐west; ExS—Extreme south; and ExN—Extreme north. The interactions were obtained through a literature survey, where each study reviewed may account for different interactions.

The literature survey conducted on pollinator–plant interactions in the Cerrado biome revealed a significant imbalance in the distribution of these interactions across biogeographic districts (Figure 5). Most interactions (89.61%) were observed in three biogeographic districts. South‐east (30.84%), Central (27.93%), and South (26.13%). On the contrary, the remaining 10.39% of interactions were spread across the South‐West (5.91%), Central‐West (2.35%), Extreme‐North (2.31%), North‐East (2.08%), Extreme‐South (1.89%), and North‐West (0.55%) districts (Figure 5).

This finding indicates a geographic bias in the current understanding of pollinator–plant interactions within the Cerrado biome. The focus of research and the available literature is disproportionately centered on the South‐East, Central, and South districts, while other districts receive relatively less attention. As a result, our knowledge of pollinator–plant interactions in the Cerrado biome is limited in certain regions, particularly the southwest, central west, extreme northwest, northeast, extreme southwest, and northwest districts.

## 4 DISCUSSION

In this study, we present a comprehensive ecological meta network of pollinator–plant interactions within the Brazilian Cerrado, which is recognized as the world's largest, most biodiverse, and threatened tropical savanna (Cardoso Da Silva & Bates, 2002). Our study reveals that the modular structure of the network aligns with findings from previous studies on partial networks in the Neotropics (Olesen et al., 2007; Schleuning et al., 2012; Souza et al., 2022; Vizentin‐Bugoni et al., 2018). Although entomophily, and particularly melittophily, is the predominant pollination syndrome in the Cerrado, other insects and vertebrates also play a crucial role in shaping the network structure. Furthermore, we showed that plants in different plant physiognomies act as connectors between modules, mediating interactions between vertebrate and invertebrate species at different periods during day and night (Souza et al., 2022).

At the meta network scale, pollination and flower–visitation interactions in the Brazilian Cerrado are predominantly governed by generalist interactions. However, based on the species composition of both animals and plants within the modules and the pollination syndromes reported in the studies, we can infer that modules were most likely influenced by trait matching due to the functional diversity on both sides of the interaction. These modules exhibit simultaneous connections of day and night species and spatial connections among plant formations found within the biome. However, it is important to acknowledge that existing knowledge about species and their interactions in the Cerrado is biased toward studies conducted in specific biogeographical districts, which experience significant intrusions from remnants of the Atlantic Rainforest biome (Françoso et al., 2020). This bias limits our complete understanding of species interactions in the Cerrado.

Bees emerged as the most influential group in terms of their contribution to family‐level interactions, aligning with previous studies conducted in the region (Martins & Batalha, 2006; Rabeling et al., 2019) and other tropical regions (Olesen & Jordano, 2002; Schleuning et al., 2012; Vizentin‐Bugoni et al., 2018). Although entomophily accounts for approximately 70% of observed pollination syndromes, bees were responsible for most insect‐mediated pollinations, comprising 55.64% of interactions. In particular, our study sheds light on the significance of diurnal lepidopterans (e.g., Nymphalidae, Peiridae, and Hesperidae) and nocturnal lepidopterans (e.g., Sphingidae, Noctuidae, and Geometridae), which are underestimated in the region (Martins & Batalha, 2006; Oliveira et al., 2004; Oliveira & Gibbs, 2000). In tropical regions, dipterans play a lesser role as pollinators compared to higher latitudes (Olesen & Jordano, 2002), as coleopterans and other insect groups exhibit a higher likelihood of being flower visitors, owing to the functional group diversity within these orders in the tropics. However, beetles are another underestimated group in the region, as the nocturnal lepidopterans.

While previous findings reporting that only 5% of Cerrado plants were pollinated by hummingbirds and bats (Martins & Batalha, 2006), our study revealed a higher prevalence of ornithophily and chiropterophily, accounting for 24.86% of the observed pollination syndromes. Also, 20.6% of the plants exclusively interacted with vertebrate species, indicating that pollination by vertebrates, especially bats and hummingbirds, is a prominent field of investigation in future studies (Lopes et al., 2021). Furthermore, many Cerrado plants exhibit mixed and diverse pollination systems involving both vertebrate and invertebrate pollinators or flower visitors (Souza et al., 2022), which may account for most pollination events in the biome. Despite the relevance of these results, they should be interpreted with caution. Literature surveys like ours may be inherently taxonomically biased through the number of published materials on specific groups of pollinators and plants, which also underestimate less frequent interactions and related pollination syndromes (Ollerton et al., 2015).

The observed modular structure in our network appears to be a common characteristic of both natural (Maruyama et al., 2014; Olesen & Jordano, 2002; Schleuning et al., 2012; Souza et al., 2022; Vizentin‐Bugoni et al., 2018) and human‐dominated landscapes (Assunção et al., 2022) in the Cerrado. Modularity is expected to increase with species richness and habitat heterogeneity, which can be explained by two mechanisms that are not mutually exclusive (Olesen et al., 2007). First, competition tends to reduce niche overlap and promote functional diversity among pollinators that have coevolved in the same environment, leading to the emergence of forbidden links that constrain species diversification and abundance (Bascompte & Jordano, 2007; Olesen et al., 2007). Second, in heterogeneous habitats, multiple foraging opportunities are available for species, thereby reducing niche overlap (Vizentin‐Bugoni et al., 2018). Therefore, modules tend to be associated with morphological specialization of animals and plants and habitat occupancy, as previously demonstrated for plant–hummingbirds networks in the Cerrado (Maruyama et al., 2018).

Although the interactions within the network display a degree of modularity, the modules of nonoverlapping species were formed by interacting species temporally separated by factors such as diurnal pollinators (e.g., bees, beetles, and hummingbirds) and nocturnal pollinators (e.g., bats, nocturnal lepidopterans) sharing the same plants (Diniz & Aguiar, 2023; Diniz et al., 2022; Souza et al., 2022), or pollinators foraging on different plant species during the dry and wet seasons (Souza et al., 2018).

The asymmetry we found in the strength of interactions and degree of specialization of plants and animals can be attributed to plants investing heavily in the production of floral rewards and structures to attract pollinators, while pollinators have a broader range of food sources and can switch between them based on availability and preference (Dalsgaard et al., 2021). This dynamic can result in a higher degree of ecological redundancy and flexibility in pollinator assemblages, leading to potential rewiring of plant–pollinator interactions and changes over time (Peralta et al., 2020). Such dynamics have significant implications for the functioning and resilience of the ecosystem in the face of environmental change.

Within the bee modules, social bees and wasps exhibit generalist behavior at the colony level, but display individual‐level specialization due to the variability in foraging behaviors between individuals within and between species, interacting with multiple generalist flowers (Pires et al., 2022). However, solitary bees exhibit more distinct differences in foraging traits. For example, solitary oil‐collecting bees (Apidae) and orchid bees (Halictidae) forage for food sources, as well as oils, resins, and perfumes for nesting (Gottsberger, 1986; Roubik, 1992). Most oil‐producing plants in the Cerrado do not produce nectar, which forces bees to forage on various plant species. These plants rely on pollinators with specialized traits for oil collection (Báez‐Lizarazo et al., 2021; Carneiro et al., 2019; Pacheco‐Filho et al., 2015) or specialized behaviors such as buzz pollination (Oliveira & Sazima, 1990; Montesinos & Oliveira, 2015; Proença & Gibbs, 1994). In addition, we have identified even more specialized interactions within the modules, such as modules primarily composed of moths, beetles, and wasps, which may rely on specialized pollination systems.

The vertebrate modules exhibit a pattern similar to the invertebrate ones, with one of the largest modules dominated by hummingbirds and another by bats. In both cases, floral traits, such as elongated flower tubes for hummingbirds and flowers with abundant exposed nectar for bats, determine which pollinators have access to the floral resources (Dalsgaard et al., 2021; Maruyama et al., 2013, 2014; Souza et al., 2022). However, recent studies suggest that these pollination syndromes may not be as strict, as bats can supplement diurnal pollination by visiting ornithophilous plants at night in the Cerrado (Diniz & Aguiar, 2023). Therefore, the diversity of traits may be more important than species diversity in explaining the differences between the network and module scales, with trait matching possibly governing the interactions that combine invertebrate and vertebrate pollination systems.

The social bees *A. mellifera* and *T. spinipes* emerged as the main species with greater strength of interaction and centrality in the network. Although *A. mellifera* is an exotic species in the Cerrado, *T. spinipes* is native (Roubik, 1992). Both species are hypergeneralists and have been associated with negative impacts on native bee assemblages in Brazil, potentially affecting the richness, abundance, and species composition of native bees (Garibaldi et al., 2021). This raises the possibility that these bee species may have been overrepresented in the sampling compared to rarer pollinator species, or that the presence of these insect pollinators threatens native insect populations in the Brazilian Cerrado biome. Hummingbirds also emerge as a central and highly connected group in our network. However, it is important to acknowledge that these findings may be influenced by a lack of attention to other vertebrate pollinators and flower‐visiting species in previous studies.

We identified a bias in the sampling of studies related to pollination and flower visitation interactions across plant physiognomies. This bias is attributable to the vast extent of the area under consideration, leading to potential variations in the selection of floral traits across species' ranges (Fenster et al., 2004). Furthermore, pollinator environments have been altered over time, which can result in divergent selection of floral traits (Anderson et al., 2014; Robertson & Wyatt, 1990), which could ultimately lead to switches in pollination systems (Diniz & Aguiar, 2023; Diniz et al., 2022). This phenomenon is particularly relevant in Cerrado, characterized by a variety of plant physiognomies including forests, savannas, and grassland‐type environments, which exhibit variations in plant species composition and provide distinct floral resources across different temporal and spatial scales (Maruyama et al., 2014; Rabeling et al., 2019). Even so, our study revealed that invertebrate and vertebrate pollinators may be constantly moving among the mosaic of vegetation types in the Cerrado. Differences in plant composition between plant physiognomies may offer different opportunities for foraging through time and provide different environmental conditions for pollinators forage and reproduce. Such traits may have contributed to the formation of modules in the network, showing that pollination events are intrinsically connected and interdependent in the landscape due to plant diversity in the biome.

The Cerrado region has been the focus of most studies, particularly in the biogeographical districts of the South‐East, Central, and South. These regions are characterized by highly fragmented landscapes and threatened native areas, primarily due to agricultural expansion (Machado & Aguiar, 2023). The intrusion of remnants of the Atlantic Rainforest is significant, particularly in the South and Southeast regions (Françoso et al., 2016). Furthermore, most universities and research centers studying pollination services are located in these regions (Ramos et al., 2020). Such a concentration of research and attention may lead to biased perceptions of pollination dynamics in the Cerrado.

Variations in plant species composition, including endemism rates, within these districts can significantly influence the occurrence of pollinators and, consequently, the network topology of the Cerrado (Françoso et al., 2016, 2020). As a result, our current network may not fully represent the diversity and evolutionary history of pollinators in the Cerrado due to the influence of biogeographical variations and regional characteristics (Dalsgaard et al., 2021; Peralta et al., 2020).

To obtain a more comprehensive understanding of pollination dynamics in this region, future research should strive to encompass a wider range of locations in less explored areas and consider the influence of different biogeographical factors. This will allow us to gain deeper insights into the complex interactions between floral traits, pollinators, and the overall network topology in the Cerrado. By incorporating these understudied areas, we can obtain a more comprehensive understanding of pollination networks throughout the region. It is also important to investigate the spatial and temporal dynamics that influence the assembly of interaction networks on a biome‐wide scale. This will enable us to identify the key factors shaping these networks and develop effective conservation strategies for the Cerrado biome and its diverse pollinator communities.

In conclusion, our study offers valuable insights into the pollinator–plant interactions within the Cerrado, the world's most biodiverse savanna by considering both invertebrates and vertebrates. The modeled network represents a significant advancement in comprehending the structural complexity of pollination in this tropical region, which is internationally recognized as a biodiversity hotspot threatened by agricultural expansion. By shedding light on the intricate structure of pollination networks, we contribute to global knowledge about ecological processes in tropical regions and the importance of conserving Earth's biodiversity hotspots. Nevertheless, network properties are highly sensitive to sampling intensity and our study represents a sample of the available studies in the subject. We must acknowledge especially the geographic bias in our sampling, which limits our complete understanding of how these interactions are truly organized in natural systems. We recommend conducting studies in understudied areas of the Cerrado to fill the knowledge gaps and unravel the spatial and temporal effects that influence interaction networks at a biome‐wide scale. This will be a crucial step toward developing effective conservation strategies for the Cerrado biome and its diverse pollinator communities.

## AUTHOR CONTRIBUTIONS

**Ludmilla M. S. Aguiar:** Conceptualization (equal); supervision (equal); writing – original draft (equal); writing – review and editing (equal). **Ugo M. Diniz:** Data curation (equal); formal analysis (equal); writing – original draft (equal). **Igor D. Bueno‐Rocha:** Data curation (equal); investigation (equal); writing – original draft (equal). **Luísa S. Aguiar‐Machado:** Data curation (equal); investigation (equal); writing – original draft (equal). **Laura R. A. Filomeno:** Data curation (equal); investigation (equal); writing – original draft (equal). **Priscilla A. Gomes:** Data curation (equal); investigation (equal); writing – original draft (equal). **Pedro H. B. Togni:** Formal analysis (equal); supervision (equal); writing – original draft (equal); writing – review and editing (equal).

## CONFLICT OF INTEREST STATEMENT

The authors have no conflict of interest.

## Supporting information


Table S1.

## Data Availability

All data sets will be also made available in a public repository such as Figshare, in case of approval of this manuscript.
